# In vitro evaluation of microfluidic WS6-loaded Eudragit nanoparticles for improving insulin-producing cell differentiation

**DOI:** 10.1371/journal.pone.0354587

**Published:** 2026-08-04

**Authors:** Duaa Abuarqoub, Marwa Mohammad, Rand Albarghouthi, Mohammad Abuoun, Mohammad Alnatour, Fuad Alhawarat, Abdolelah Jaradat

**Affiliations:** 1 Faculty of Pharmacy and Medical Sciences, University of Petra, Amman, Jordan; 2 Department of Applied Medical Sciences, Al Hussein Bin Abdullah II Academy for Civil Protection, Al-Balqa Applied University, Salt, Jordan; 3 Department of Applied Pharmaceutical Sciences and Clinical Pharmacy, Faculty of Pharmacy, Isra University, Amman, Jordan; Università degli Studi della Campania, ITALY

## Abstract

This study explores a stem-cell–based approach for diabetes treatment by enhancing the viability and functionality of insulin-producing cells (IPCs) derived from stem cells of the apical papilla (SCAP). Although SCAP can differentiate into IPCs, limited cell survival remains a challenge. To address this, the proliferation enhancer WS6 was incorporated into Eudragit RS100 nanoparticles (NPs) using microfluidics. The WS6-loaded NPs were characterized for size, charge, PDI, morphology, stability, and drug loading. An MTT assay was performed as a preliminary screening method to evaluate the cytocompatibility of blank-NPs and to optimize treatment concentration. SCAP cells were treated with free WS6 or WS6-loaded NPs, and cellular uptake of NPs was evaluated using flow cytometry and fluorescence imaging. Additionally, the viability of treated cells was determined by propidium iodide (PI) and trypan blue. Prior to differentiation, definitive endoderm formation was assessed through SOX17 and FOXA2 expressions. After differentiation into IPCs, maturation markers such as insulin, C-peptide, PDX-1, NKX2.2, and NKX6.1 were examined, and apoptosis assays measured cell viability. Functional insulin secretion was tested using an in vitro glucose-stimulated insulin secretion (GSIS) assay. Results showed that WS6-loaded NPs significantly improved SCAP viability, increased healthy cell percentages, and enhanced IPC maturation. Treated IPCs demonstrated functional insulin secretion and improved glucose regulation. Overall, WS6-loaded NPs represent a promising approach to enhance IPC proliferation and generation for diabetes therapy.

## 1. Introduction

Diabetes Mellitus (DM) is a global public health burden; approximately 585 million people are living with diabetes according to the last report of the International Diabetes Atlas (IDF) in 2025 [1]. The chronic hyperglycemia of diabetes is associated with long-term damage, dysfunction, and failure of various organs [2]. Promoting islet β‐cell regeneration and restoring endogenous insulin secretion may present an ideal approach to curing diabetes [3–5]. Yet, efforts to engineer islet-like cells or insulin-producing cells from different types of stem cells have offered an appealing alternative to islet transplants. Stem cell therapy avoids some of the serious drawbacks of islet transplantation, most obviously, the shortage of organ donors. As with most organ transplants, a limiting factor for islet transplantation is supply [6]. Stem cells exhibit multi-directional differentiation potential, enabling them to differentiate into insulin-producing β-cells (IPCs) under specific induction conditions. These differentiated β-cells closely resemble their natural counterparts in both structure and function, responding to fluctuations in blood glucose and secreting appropriate insulin amounts to regulate glucose homeostasis [7].

Therefore, the versatile properties of mesenchymal stem cells (MSCs) have generated their clinical interest as therapies for diabetes [8]. MSCs derived from dental apical papilla tissue (SCAP) are known for their differentiation potential into different lineages [9]. SCAP shows a great potential to differentiate into functional insulin-producing β-cells, thereby offering a promising approach for β-cell replacement [10]. In addition to their differentiation capacity, stem cells are known to support surrounding cells through multiple mechanisms, including metabolic modulation and intercellular organelle transfer. Recent studies have demonstrated that stem cell-mediated mitochondrial transfer can influence cellular survival and stress responses, highlighting the role of stem cells in maintaining cell viability and functionality within challenging microenvironments [11]. Despite these advances, challenges such as low survival rates of transplanted cells and limited insulin-secreting capacity remain unexplored. Furthermore, the use of IPC in clinical applications requires consideration of the pathological microenvironment in diabetes, particularly within pancreatic islets. A significant alteration in the islet microvasculature has been reported, including endothelial dysfunction, basement membrane thickening, and pericyte loss. These structural and functional changes impair oxygen and nutrient delivery, thereby compromising cell survival and insulin secretion capacity. In addition to vascular impairment, the inflammatory microenvironment characteristic of insulitis represents a major barrier in diabetes. Insulitis is characterized by immune cell infiltration, local release of pro-inflammatory cytokines, and progressive β-cell destruction [12]. Such inflammatory and vascular factors significantly influence cell survival and function. Therefore, IPCs used in therapy must overcome both immune-mediated damage and microenvironmental stress [11]. Recently, stem cell therapy has extended from 2D differentiation systems toward more complex 3D culture and organoid-based approaches, which may better mimic the native pancreatic microenvironment and improve cell survival and functionality following transplantation [13]. Accordingly, agents that stimulate human pancreatic beta cell proliferation are needed to improve diabetes mellitus treatment; therefore, various types of small molecules have been used to induce the proliferation rate of derived beta cells, consequently increasing their survival rate. Among the small molecules identified for stimulating β-cell proliferation, WS6 has shown considerable promise.WS6 is a diarylamide compound identified through high-throughput screening using R7T1 rat β-cell lines and has been reported to induce proliferation in both rat and human β-cells in a dose-dependent manner [14]. Its mechanism of action involves modulation of the IκB kinase (IKK) pathway and inhibition of ErbB3-binding protein 1 (EBP1/PA2G4), key players in NF-κB signaling and cell cycle regulation [14,15].WS6 does not induce differentiation but specifically promotes β-cell and even α-cell proliferation, making it a unique agent in regenerative strategies [16]. Importantly, WS6 has also demonstrated the ability to enhance cell viability, a critical factor for the success of extending IPCs’ lifespan as a therapeutic approach. Studies have shown that WS6 treatment improves β-cell survival, increases cell numbers, and helps in normalizing blood glucose levels in diabetic models [17]. When incorporated into nanoparticle delivery systems, WS6’s efficacy is further enhanced through sustained release and targeted delivery to differentiated cells, potentially improving their insulin secretion and therapeutic lifespan. Polymer-based nanoparticles (NPs) can encapsulate and protect bioactive molecules to enable sustained release and enhanced therapeutic efficacy in targeted tissues, including the pancreas. Eudragit RS100 is a non-toxic, biocompatible, and time-dependent synthetic polymer that enables sustained drug release through its permeability, which is regulated by quaternary ammonium groups [18,19], all combined, possibly promoting proliferation [20].

Eudragit RS100 has been successfully engineered for drug delivery, with increasing potential in stem cell modulation and regenerative applications, because of its biocompatibility and its sustained release profile [20]. Given these advantages, combining WS6 with Eudragit RS-based NPs represents a novel and synergistic approach for enhancing the viability and function of stem cell-derived IPCs. This strategy holds strong potential for the development of effective, non-invasive treatments for diabetes, capable of overcoming current limitations in cell therapy and improving clinical outcomes by extending the lifespan of IPCs. Hence, we aimed in this study to prepare Eudragit RS NP s loaded with WS6, and study their impact on the IPCs derived from SCAP, and explore the effect of WS6 on the differentiation and proliferation of the derived IPCs.

## 2. Materials and methods

### 2.1. Preparation of WS6-loaded nanoparticles (WS6-NPs)

WS6-loaded NPs were synthesized using a microfluidic system (Dolomite Microfluidics, UK). As previously described [21]. The microfluidic chip was a quartz-based X-junction design with dimensions of 22.5 × 15 × 4 mm, and the channel width was 190 µm. Eudragit-RS dissolved in acetone (1 mg/ml) and loaded with WS6 (284.3 µg), then introduced into the central phase at 0.25 ml/min. Simultaneously, an acetate buffer (pH 5) was infused through the lateral phase at 0.5 ml/min, maintaining dual-phase flow for 2 min. The collected solution was stirred at 150 rpm to evaporate acetone, next a purification via centrifugation (2000xg, 5mins) was done using an Amicon tube (cutoff 100KD) with 3 times of PBS washes. Finally, the sterilization process was done under UV light for 20 min.

To ensure removal of free WS6, NPs were washed twice with PBS using Amicon ultrafiltration tubes. Since the efficiency of encapsulation was 55%, approximately 45% of WS6 remained unencapsulated initially. After each washing/concentration cycle (10 mL → 1 mL), only 10% of the free unloaded amount remained. Therefore, the residual free WS6 after two washing steps was theoretically calculated to be less than 1%.

### 2.2. Characterization of WS6-loaded NPs

#### 2.2.1. In-vitro stability Study by Dynamic Light Scattering (DLS).

For the stability study, WS6-NPs were diluted in cell culture media and incubated at 37°C for 24, 48, and 72hrs. At each time point, WS6-NPs were analyzed for size, polydispersity index (PDI), and zeta potential (n = 3). The latter-mentioned physical properties of WS6-loaded NPs were assessed using dynamic light scattering (DLS) (Zetasizer, Malvern Instruments Ltd., UK). For analysis, 50 µL of WS6-loaded NPs was diluted in 950 µL of distilled water prior to measurement [21] (Altmann, Portela et al. 2025).

#### 2.2.2. WS6 Content measurement.

WS6 content of loaded NPs was measured using an indirect RP-HPLC method (Thermo Scientific™, USA) following the literature [22]. The mobile phase consisted of methanol and ammonium acetate (10 mM, pH 7.8) (80:20, v/v). WS6-loaded NPs were analyzed at a flow rate of 1 ml/min using a C18 column (4.6 mm × 150 mm, 5 µm; Agilent, US). WS6 was detected at 252 nm using a UV detector, with a retention time of 3.8 min. WS6 was diluted 1:1 in mobile phase and analyzed via HPLC. The EE% was calculated using the following equation. All procedures followed Tocris Bioscience protocols with minor modifications.


$$\begin{array}{c} \text{Encapsulation efficiency }\%=\frac{\text{Total amount of WS6 }-\text{ the amount of unentraped WS6}}{\text{Total amount of WS6 }}\;\times \text{100}\% \end{array}$$

### 2.3. Cell culture

The study was conducted in accordance with the guidelines of the Declaration of Helsinki. It was approved by the Institutional Review Board of the Cell Therapy Center at The University of Jordan (IRB-CTC/1-2023-04), effective as of 12 February 2023. Informed consent was obtained in writing from all participants involved in the study. Sample recruitment began on April 1, 2023, and concluded on December 1, 2024. For participants under the age of 18, written informed consent was acquired from their parents or legal guardians before participation.

SCAP cells were extracted from impacted third molars as previously described [23]. The derived cells were obtained and approved by the institutional review board (IRB) at the Cell Therapy Center/University of Jordan (IRB-CTC/1-2023-04). SCAP were cultured in α-MEM supplemented with 10% fetal bovine serum (FBS), 1% penicillin-streptomycin, and 1% L-glutamine. Cells were maintained at 37°C in a humidified incubator with 5% CO_2_, with medium changes every 2–3 days. Upon reaching 80–90% confluency, cells were passaged using 1x trypsin-EDTA [10].

#### 2.3.1. Dose-Response curve (MTT assay).

SCAP cells were seeded in triplicate in a 96-well plate (SPL Life Sciences, Pocheon, Korea) at a density of 2500 cells/well for 72hrs of treatment. Cells were then treated with WS6, WS6-loaded NPs, and blank NPs at concentrations ranging from 0.125 to 4 µM using 2-fold serial dilutions. Cell viability was evaluated using the MTT assay (Abcam, Cambridge, UK), and absorbance was measured at 590 nm with a microplate reader (Thermo Fisher Scientific, Waltham, MA, USA).

### 2.4. Cellular uptake

#### 2.4.1. Coumarin-6 labelling of WS6- loaded NPs.

WS6-loaded NPs were labeled using coumarin-6 (C6) fluorescent dye (Sigma, USA) at a concentration of 0.05 mg/ml. C6 was incorporated into the central phase along with Eudragit-RS and WS6, then the NPs were prepared as described earlier in the methodology [24].

#### 2.4.2. Confocal imaging.

Cellular uptake of WS6-loaded NPs was evaluated using a confocal microscope (Zeiss, Germany). SCAP were seeded on coverslips, then treated with C6-labeled WS6-loaded NPs for 24hrs. Following treatment, cells were fixed with 4% paraformaldehyde (PFA), (GHD, Guangdong Guanghua Chemical Factory Co. Ltd, China) for 15 mins, then washed with PBS. DAPI (4′,6-diamidino-2-phenylindole) (Themo Scientific, Germany) (diluted at 1:1000 in distilled water) was added and incubated for 15 mins in the dark, then washed with PBS. Finally, 20 µL of mounting media (DAKO, Santa Clara, CA, USA) was applied onto a microscope slide for imaging [24].

#### 2.4.3. Flow cytometry.

As previously described for the uptake study, after seeding and treating SCAP with C6-labeled WS6-loaded NPs for 24hrs, cells were harvested by trypsin-EDTA, washed with PBS, and introduced into flow cytometry, and C6-labeled NPs were detected through a FITC filter [25].

### 2.5. Viable/dead cells discrimination

A viable/dead cell discrimination assay was performed using two methods: Propidium Iodide (PI) and trypan blue staining.

#### 2.5.1. PI staining.

Briefly, cells were seeded with 50*10^3^ per well and treated with free WS6 and WS6-loaded NPs (1µM) for 24hrs and 72hrs. Next, cells were harvested and stained with (10 µg/ml) Propidium Iodide stain (PI) (Abcam, UK) in PBS, then incubated in the dark at room temperature for 15 mins. The samples were analyzed using BD Accuri™ C6 Plus Personal Flow Cytometer (BD Biosciences, USA) [26].

#### 2.5.2. Trypan blue staining.

For trypan blue staining, SCAP cells treated with free WS6 and WS6-loaded NPs (1µM) were collected and stained with trypan blue (Gibco, USA), then counted by hemocytometer and observed under the microscope, following 24hrs and 72hrs of treatment [27].

### 2.6. IPCs differentiation

As previously described [23], IPCs differentiation started with the initiation of the definitive endoderm layer. SCAP were seeded in 12-well plates with 30*10^3^ cells per well. Next, α-MEM was replaced with definitive endoderm (DE) induction media, and supplements were added as instructed by the manufacturer (Stem Xvivo, Endoderm Kit, R&D system, UK) for 3 days. From day 4 until day 7, media was replaced with serum-free advanced DMEM-F12 (Thermofisher, Waltham, MA, USA), supplemented with 1% BSA (Bovine serum albumin, Biowest, Nuaillé, France), 1% ITS (Insulin Transferrin selenium, Sigma, Burlington, MA, USA), 0.3 mM Taurine (Sigma, Burlington, MA, USA) to induce pancreatic progenitor stage. For the differentiation into functional pancreatic cells, the media was exchanged by adding serum-free advanced DMEM-F12 supplemented with 1.5% BSA, 1.5% ITS, 3 mM Taurine, 100 nM GLP (glucagon-like peptide Sigma, Burlington, MA, USA), and 1 mM nicotinamide (Sigma, Burlington, MA, USA at day 8 of differentiation until day 20). On day 21, differentiated cells were treated with 1µM of free WS6 and WS6-loaded NPs for 24hrs and were compared to the differentiated WS6-free groups.

#### 2.6.1. Validation of definitive endoderm markers.

**2.6.1.1. Flow cytometry:** To measure the expression levels of endodermal markers (FOXA2 and SOX17), differentiated cells were collected at day 7 of induction by trypsinization, then fixed and permeabilized with methanol, and then stored at −20°C until use. Afterward, the cells were washed with PBS and stained with SOX17-APC (BD Pharmingen™ Alexa Fluor® 647 mouse anti-human SOX17) and FOXA2 (BD Pharmingen™ PE mouse anti-human FOXA2). Cells were stained by adding 1:10 diluted antibody in stain buffer (BD Biosciences, USA) for 30 mins incubation. The cells were centrifuged and washed with PBS, and finally, the samples were analyzed by using BD Accuri™ C6 Plus Personal Flow Cytometer (BD Biosciences, USA). Cells collected on day 1 of induction were used as an internal control for normalization. For sample acquisition, 10,000 events were acquired per sample. A gated population of cells was selected as the gate (P1). The mean fluorescence intensity of each marker within the gated population was analyzed and plotted in a histogram.

**2.6.1.2. SOX17 -Immunofluorescence staining (IF):** The expression of the SOX17 marker was evaluated by using the StemXvivo endoderm Kit (R&D Systems, UK) and visualized under the fluorescent confocal microscope (Zeiss, Germany) using AF555 filter and DAPI filter [28].

#### 2.6.2. Validation of IPCs maturation markers.

**2.6.2.1. Validation of maturation markers by immunofluorescence (IF):** At day 23 of differentiation, differentiated cells were examined to determine the maturity state of the induced β-cells by measuring the expression of the following maturation markers: insulin, c-peptide, PDX-1, NKX6.1, and NKX2.2. Differentiated cells were fixed with 4% paraformaldehyde for 20 mins, permeabilized with (0.25% triton + 0.1% BSA, Sigma-Aldrich®, USA) for 20 mins, followed by a further step of blocking for 20 mins by adding the blocking solution: 0.1% triton+ 0.5% goat serum (Euroclone S.p.A., Italy) + 1% BSA in PBS. Following that, cells were washed with PBS then, antibodies were added and incubated overnight at 4 °C as follows; Insulin (Abcam, UK) 1:200 (host: rabbit), c-peptide (Abcam, UK) 1:100 (host: rabbit), PDX-1 (Abcam, UK) 1:1000 (host: rabbit), NKX2.2 (R&D, biotechne, USA) diluted as 8 µg/ml (host: mouse), and NKX6.1 (R&D, biotechne, USA) diluted as 8 µg/ml (host: mouse). Next, cells were washed with PBS and incubated with secondary antibodies: anti-rabbit IgG Alexa Flour^TM^ plus 488 and anti-mouse IgG Alexa Flour^TM^ 546 at a 1:1000 dilution for 1 hr in the dark at room temperature. After that, cells were incubated for 15 minutes with DAPI (4′,6-diamidino-2-phenylindole) for nuclear staining. Then, coverslips were flipped on a glass slide and mounted with 10µl mounting media (DAKO, USA). Finally, cells were observed using a confocal microscope (Zeiss, Oberkochen, Germany) by using AF488 filter, AF555 filter, and DAPI filter [29].

**2.6.2.2. Measurement of insulin release by mature IPCs** At the end of differentiation, the supernatant (media) was collected from the IPCs treated with free WS6, WS6-NPs, and blank-NPs and stored at −20°C until plate preparation. The assay was accomplished using the Human Insulin ELISA (Enzyme linked immunosorbent assay) Kit (R&D Systems™, UK). Optical density was determined using a microplate reader (Thermo Scientific™, USA) set at 450 nm and corrected at 540 nm. The secretion level of insulin was normalized to the cell count of each treatment group.

#### 2.6.3. Cell death modality after differentiation (apoptosis/necrosis assay).

At the last stage of differentiation (day 23), treated cells with free WS6 and WS6-loaded NPs were harvested, washed then stained with annexin V and PI as instructed by the manufacturer (Invitrogen kit, USA). Samples were analyzed using BD Accuri™ C6 Plus Personal Flow Cytometer (BD Biosciences, USA) [30].

#### 2.6.4. Functional assay.

**2.6.4.1 Glucose-stimulated insulin secretion assay (GSIS) in vitro:** At the end of differentiation, IPCs treated with 1 µM free WS6 and WS6-loaded NPs were subjected to a glucose challenge GSIS assay. Cells were washed and preincubated in Krebs buffer containing 2 mM glucose for 1hr to remove residual insulin. Two glucose challenge cycles were performed, each consisting of 1hr incubation in low-glucose (2.8 mM) followed by 1hr in high-glucose (10 mM), with media collection after each step. Finally, cells were incubated in Krebs buffer containing 2 mM glucose and 30 mM KCl for 30 mins for depolarization. Insulin levels in the collected media were measured using a human Insulin ELISA kit (R&D Systems™, UK), and optical density was read using a microplate reader at 450 nm (Thermo Scientific™, USA). The secretion level of insulin was normalized to the cell count of each treatment group, as previously described [31,32].

### 2.7. Statistical analysis

All experiments and measurements were performed in triplicate(N = 3), and results were reported as mean ± standard deviation (Mean ± SD) for each experiment. Comparisons between multiple groups were performed by Two-way ANOVA for the following experiments: apoptosis assay after differentiation, viable-dead discrimination assays stained with PI, and quantification of insulin released by ELISA. For cell counting, one-way ANOVA was used, followed by Tukey’s multiple comparison test. All statistical analyses were performed in GraphPad Prism 8.0. 1. P-values < 0.05 were considered significant.

## 3. Results

### 3.1. Characterization of WS6-loaded NPs

#### 3.1.1. Stability results: DLS and content measurement.

WS6-NPs stability was evaluated over 72 hrs, and results showed that particle size distribution was stable throughout the study, with mean diameters of 149.9 ± 27.7 nm, 157.3 ± 7.4 nm, and 141.0 ± 22.0 nm at 24, 48, and 72hrs, respectively. No significant aggregation was observed compared to freshly prepared WS6-NPs 149.3 ± 15.2 nm.

**Fig 1:**
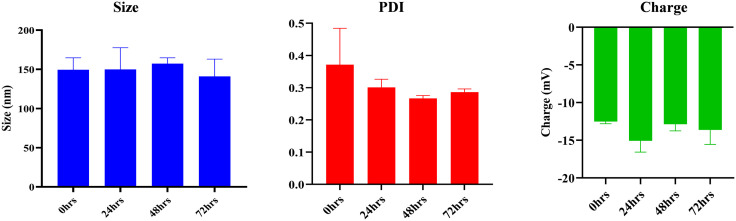
In vitro stability of WS6-NPs over 72hrs in culture media at 37 °C compared with freshly prepared nanoparticles(0hrs), by dynamic light scattering (DLS), including: A) particle size, polydispersity index (PDI), and charge (zeta potential). (N = 3). Data are presented as Mean±SD.

Similarly, the PDI values remained below 0.3 across the incubation period, indicating good dispersion stability. The values were 0.301 ± 0.025, 0.267 ± 0.009, and 0.286 ± 0.010 at 24, 48, and 72hrs, respectively. In addition, zeta potential measurements showed mean surface charges of −15.077 ± 1.49 mV, −12.879 ± 0.879 mV, and −13.62 ± 1.939 mV at 24, 48, and 72hrs, respectively, indicating overall colloidal stability. The observed shift toward a slightly negative charge upon incubation in cell culture media may be attributed to the adsorption of serum proteins and other media components onto the nanoparticle surface. Overall, these results demonstrate that WS6-NPs maintain good physicochemical stability over 72hrs when compared to freshly prepared nanoparticles (Fig 1).

### 3.2. Dose-Response curve (MTT assay)

**Fig 2:**
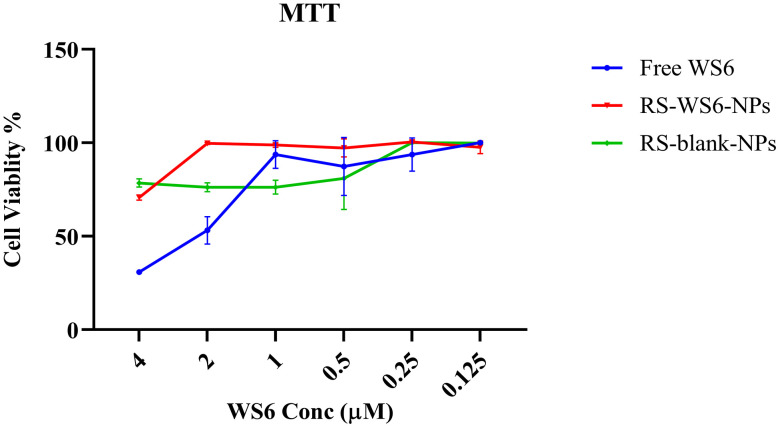
Dose–response curves (MTT assay) of stem cells from the apical papilla (SCAP) treated with free WS6, WS6-loaded(WS6-NPs), and equivalent concentrations of blank-NPs among a concentration range of 0.125–4 µM for 72hrs. Statistical analysis was performed using two-way ANOVA followed by Dunnett’s multiple comparison test (N = 3). Data are presented as mean ± SD.

MTT cell viability results showed a pronounced effect of the treatment, following 72 hrs of treatment. Free WS6 group demonstrated an obvious dose-dependent reduction in cell viability, particularly at higher concentrations (2–4 µM). For the free WS6-treated group, the viability was 93.735 ± 7.361% at 1 µM, 53.135 ± 7.29% at 2 µM, and 30.835 ± 0.559% at 4 µM. In comparison, WS6-NPs showed higher viability at corresponding concentrations, with cell viability of 98.767 ± 1.179% at 1 µM, 99.667 ± 0.557% at 2 µM, and 70.690 ± 1.4% at 4 µM, while blank-NPs exhibited relatively low cytotoxicity through these concentrations, with cell viability of 76.24 ± 3.649%, 76.19 ± 2.319%, and 78.47 ± 2.178% at 1, 2, and 4 µM, respectively. Overall, WS6-NPs demonstrated improved biocompatibility compared with free WS6, maintaining higher cell viability among all used concentrations and exposure period (Fig 2)

### 3.3. Uptake study

**Fig 3:**
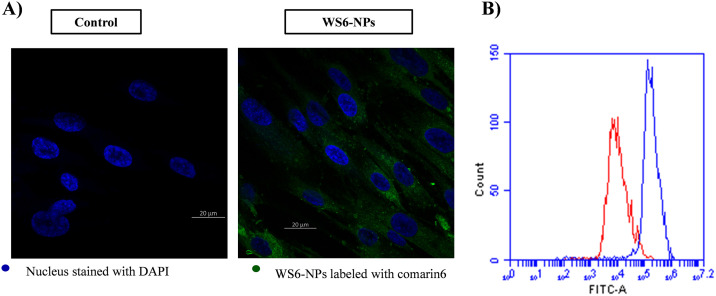
Cellular uptake of WS6-loaded NPs labelled with coumarin 6 by stem cells from the apical papilla (SCAP) confirmed by: A) Confocal microscopy images, Green (Coumarin 6), Blue (DAPI). Confocal images were acquired at 63 × magnification. By using DAPI filter and FITC filter, and (B) flow cytometry histograms showing SCAP cells treated with Coumarin 6-labeled WS6-loaded NPs (1 µM) for 24hrs compared to untreated controls.

For the uptake study, our results confirmed the successful uptake of C-6 labeled WS6-loaded NPs by the SCAP cells treated for 24hrs with 1µM WS6-loaded NPs by confocal microscopy and flow cytometric analysis. The results showed that the uptake percentage was up to 61%, compared to the control untreated cells, as shown in Fig 3.

### 3.4. Viable/dead cells discrimination

#### 3.4.1. PI staining.

The effects of 1 µM of free WS6 and WS6-loaded NPs on the viability of treated cells were examined. The data indicated that free WS6 caused a significant decrease in the percentage of viable cells (p < 0.05) compared to the control group. The results for the percentage of viable cells were as follows: WS6-NPs: 91.53 ± 1.28, Free WS6: 81.30 ± 3.85, Control: 92.23 ± 0.71. Conversely, the number of propidium iodide-positive (PI + ve) cells was as follows: WS6-NP: 8.43 ± 1.28, Free WS6: 13.69 ± 0.85, Control: 7.76 ± 0.74. Remarkably, at 24hrs, free WS6 showed a significantly higher percentage of (PI + ve) cells compared to the control untreated, indicating rapid and early cytotoxicity of the free WS6. In contrast, WS6-NPs exhibited lower cytotoxicity, suggesting a protective and sustained release effect.

**Fig 4:**
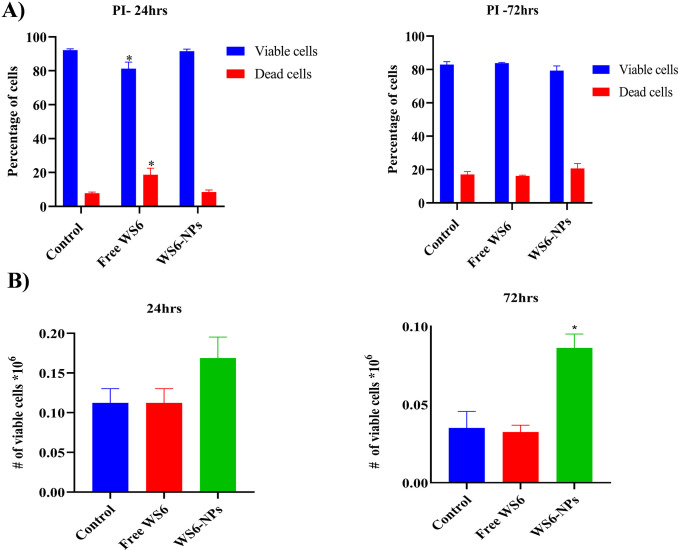
Viable/dead cells discrimination by: A) flow cytometry analysis of percentage of viable and dead cells stained with propidium iodide (PI), B) Cell count by trypan blue, of stem cells from apical papilla (SCAP) treated with 1 µM of free WS6 and WS6-loaded NPs for 24hrs and 72hrs. A two-way ANOVA test was used for statistical analysis for PI staining and a one-way ANOVA for cell counting (* < 0.05). (N = 3).Bars represent means ±SD.

After 72hrs, no significant changes were observed; the number of PI + ve cells was WS6-NPs: 20.74 ± 2.85, Free WS6: 16.21 ± 0.42, Control: 17.03 ± 1.03, with the WS6-NPs treated group being slightly higher but with no statistical differences (p > 0.05). The percentages of viable cells after 72hrs were WS6-NPs: 79.25 ± 2.86, Free WS6: 83.78 ± 0.43, and Control: 82.79 ± 1.76 (Fig 4A).

#### 3.4.2. Trypan blue staining (Cell counting).

The effects of 1 µM free WS6 and WS6-loaded nanoparticles on cell count were evaluated. Results indicated a slight increase in the cell count for the WS6-loaded nanoparticle (NP) group after 24hrs compared to the control group. The cell counts were as follows: (WS6-NPs: 0.169 × 10^6 ± 0.27, Free WS6: 0.113 × 10^6 ± 0.06, Control: 0.113 × 10^6 ± 0.27).

After 72hrs of treatment, cells treated with WS6-loaded NPs exhibited a significant increase in the number of viable cells (p < 0.05) compared to those treated with free WS6 and the untreated control groups. The cell counts at this time point were as follows: (WS6-NPs: 0.09 × 10^6 ± 0.27, Free WS6: 0.03 × 10^6 ± 0.00, Control: 0.03 × 10^6 ± 0.01) (Fig 4B).

### 3.5. Differentiation

#### 3.5.1. Validation of definitive endoderm markers: SOX17 and FOXA2.

**Fig 5:**
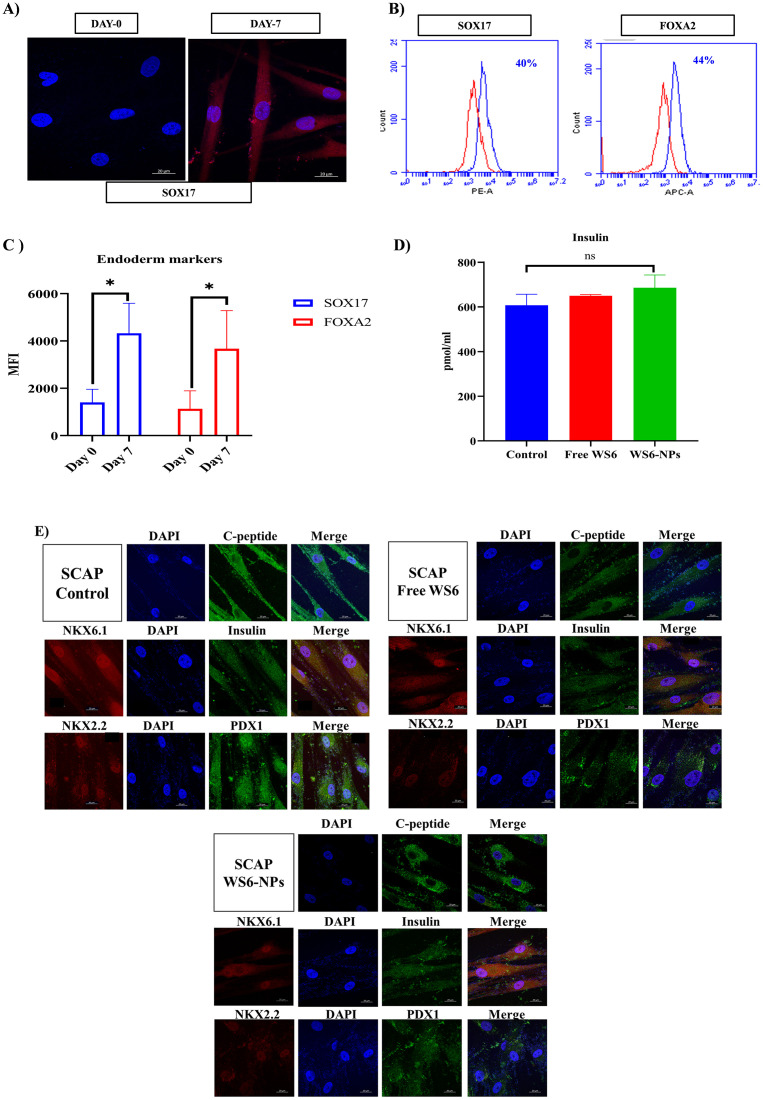
IPCs differentiation. Definitive endoderm markers validation by: A) Confocal microscope imaging showing SOX17 expression of stem cells from the apical papilla (SCAP), B) Flow cytometric histograms, and C) The chart represents mean fluorescent intensity (MFI), indicating the expression levels of SOX17 and FOXA2 in induced SCAP differentiated compared to day 1 of induction. SOX17 and FOXA2 were detected using PE and APC flow channels, respectively. D) Quantification of insulin release after 22 days of differentiation by ELISA. E) Immunofluorescence staining for the differentiation markers (Insulin, C-peptide, PDX-1, NKX6.1, NKX2.2) after 22 days of differentiation of control differentiated untreated stem cells from apical papilla (SCAP) and treated with free WS6 and WS6-loaded NPs for 24hrs. Images were taken using a confocal fluorescent microscope (63X magnification, scale bar length = 20µm). Red: NKX6.1 and NKX2.2 signal, Green: Insulin, PDX-1, and c-peptide signal. Blue: DAPI-Nucleus. Bars indicate mean ±SD. (*p < 0.05). Statistical analysis was performed using one-way ANOVA followed by Dunnett’s multiple comparison test (N = 3). Data are presented as mean ± SD.

Our results confirmed that by day 7 of differentiation, the induced SCAP cells exhibited an approximately 41% increase in the expression levels of SOX17 and FOXA2 compared to undifferentiated cells (day 1). For SOX17, the expression level was detected by fluorescent microscopy, and a significant increase in the expression of both markers was observed using flow cytometry (p < 0.05) as illustrated in the accompanying data (Fig 5A-C).

#### 3.5.2. Quantification of insulin release by ELISA.

For the quantification analysis of insulin, our data showed that cells treated with WS6-NPs showed a remarkable increase in the secreted level of insulin compared to the cells treated with free WS6 and blank-NPs. However, no statistically significant difference was shown among all treated groups as illustrated in Fig 5D.

#### 3.5.3. Validation of maturation markers by confocal microscopy.

The maturation of islet-like clusters was evaluated by examining the expression of the maturation markers: Insulin, c-peptide, PDX-1, NKX6.1, and NKX2.2 using a confocal microscope.

Following a 22-day differentiation, then 24hrs of treatment with free WS6 and WS6-loaded NPs, mature insulin-producing cells (IPCs) derived from SCAP were observed to express all mentioned markers among free WS6-treated and WS6-loaded NPs groups. It indicates that WS6 maintains the differentiation and maturation capability of these cells to differentiate into IPCs (Fig 5E).

#### 3.5.4. Cell death modality after differentiation (apoptosis/necrosis assay).

The effect of free WS6 and WS6-loaded nanoparticles (NPs) on the viability of treated cells was examined from day 21 to day 22 of differentiation. The results indicated a significant increase in the number of healthy cells in the WS6-loaded NP treated group compared to the control group, the differentiated untreated group, and the group treated with free WS6 (p < 0.05). The percentages of healthy cells were as follows: WS6-NP: 70.18 ± 0.45, Free WS6: 59.38 ± 0.40, and Control: 64.65 ± 0.78.

**Fig 6:**
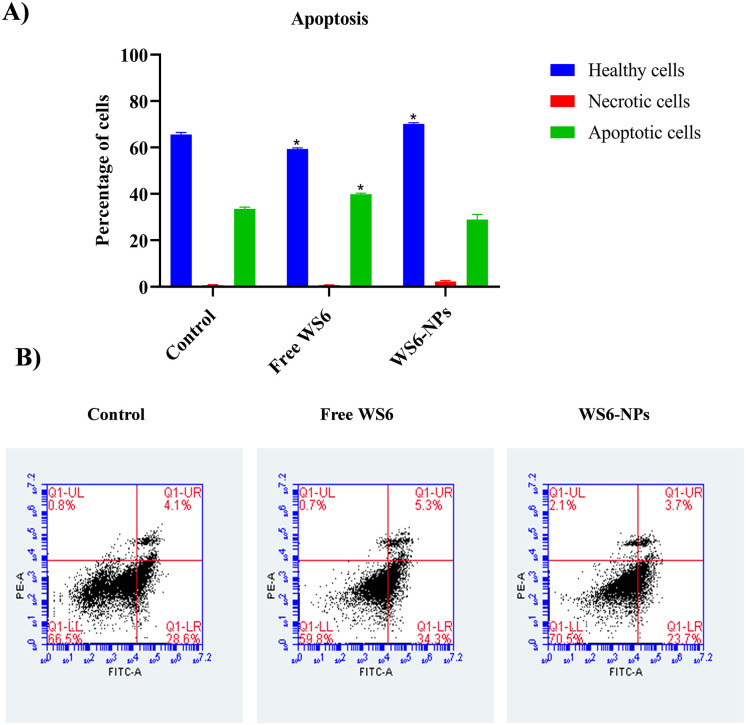
Flow cytometric analysis of percentages of healthy and (apoptotic/necrotic) stem cells from apical papilla (SCAP) treated with 1 µM of free WS6 and WS6-loaded NPs at day 22 of differentiation for 24hrs and compared to the control (differentiated untreated group). A two-way ANOVA test was used for statistical analysis. Bars represent means±SD. B) Flow cytometric dot plots of apoptosis/necrosis assay of treated SCAP for 24hrs compared to untreated control. The following quadrants represent: Q1-UL (upper left): Necrosis, Q1-UR (upper right): Late apoptosis, Q1-LL (lower left): Healthy cells and Q1-LR (lower right) Early apoptosis. Statistical analysis was performed using two-way ANOVA followed by Dunnett’s multiple comparison test (N = 3). Data are presented as mean ± SD (*p < 0.05).

Additionally, cells treated with free WS6 demonstrated a significant decrease in the number of healthy cells (p < 0.05). Unexpectedly, there was also a significant increase (p < 0.05) in the percentage of apoptotic cells in the free WS6-treated group compared to both the WS6-loaded NPs and the control untreated group. The percentages of apoptotic cells were as follows: WS6-NP: 28.92 ± 2.21, Free WS6: 39.81 ± 0.40, and Control: 33.50 ± 0.76. The percentages of necrotic cells were recorded as follows: WS6-NP: 2.27 ± 0.32, Free WS6: 0.73 ± 0.07, and Control: 0.63 ± 0.23 (Fig 6).

### 3.6. GSIS: Quantification of insulin in response to glucose challenge released by ELISA

**Fig 7:**
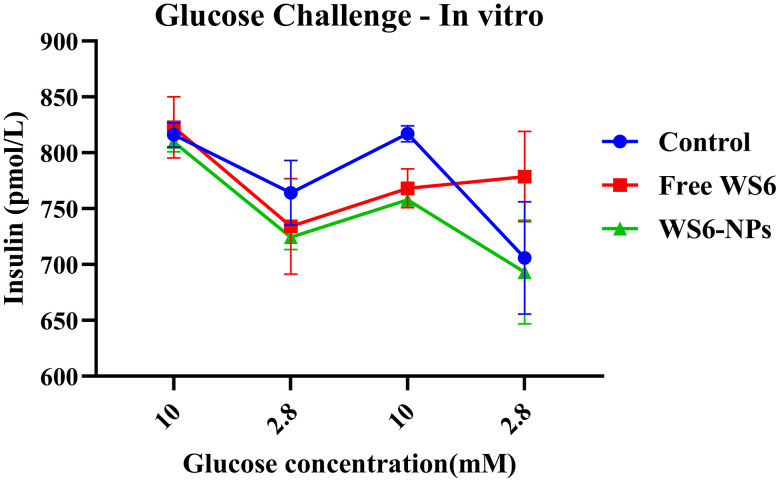
Glucose challenge analysis: *In vitro*: Insulin secretion by induced SCAP cells treated with 1µM of free WS6 and WS6-loaded NPs for 24hrs quantified by ELISA after low (2.8 mM) and high (10 mM) glucose cycles. The insulin secretion level was normalized to the cell count of each treatment group. Statistical analysis was performed using two-way ANOVA followed by Dunnett’s multiple comparison test (N = 3). Data are presented as mean ± SD.

For insulin quantification, the glucose challenge showed that IPCs treated with free WS6 and WS6-loaded nanoparticles (NPs) exhibited a clear insulin response profile with an insulin stimulation index (SI) (secreted insulin at high glucose condition/ secreted insulin at low glucose condition) greater than 1, reflecting changes in glucose levels during the differentiation protocol. In the first cycle with high-glucose conditions (10 mM), insulin secretion significantly increased, with measured concentrations as follows: WS6-NP: 809.50 ± 8.71, Free WS6: 822.80 ± 27.30, and Control: 816.09 ± 10.85 pmol/L. Conversely, during low-glucose conditions (2.8 mM), insulin concentrations decreased, yielding values of WS6-NP: 724.30 ± 10.93 (SI = 1.119 ± 0.005), Free WS6: 734.21 ± 40.73 (SI = 1.117), and Control: 764.18 ± 28.80 (SI = 1.068) pmol/L. For the second cycle at 10 mM of glucose, insulin concentrations were as follows: WS6-NP: 757.95 ± 3.33, Free WS6: 768.23 ± 17.34, and Control: 817.05 ± 7.09 pmol/L. In low-glucose conditions (2.8 mM), insulin concentrations were: WS6-NP: 693.24 ± 46.31 (SI = 1.093), Free WS6: 778.74 ± 40.42 (SI = 0.987), and Control: 705.80 ± 50.25 (SI = 1.158) pmol/L. Overall, these results showed consistent functional β-cell behavior across both glucose challenge cycles (Fig 7).

## 4. Discussion

Recent progress in stem cell therapy has enabled the differentiation of mesenchymal stem cells (MSCs) into insulin-producing cells (IPCs), yet the limited viability and short lifespan of the differentiated cells continue to restrict their therapeutic potential [33].

To overcome restrictions, small compounds have been used to improve the survival of beta cells by stimulating their proliferation. WS6 has shown significant promise in this context, enhancing β-cell survival, increasing cell numbers, and normalizing blood glucose levels in diabetic animals [15]. Its effectiveness is further boosted when delivered through nanoparticle systems, which ensure continuous release and targeted distribution to the cells, potentially enhancing insulin secretion and therapeutic longevity.

To the best of our knowledge, this study is the first to investigate suing of NPs as WS6 delivery approach for IPCs. In contrast to a prior study that examined the impact of WS6 in its free form on human islet cell survival, proliferation, and beta cell differentiation [14].

Initially, WS6 was encapsulated into polymeric NPs using Eudragit RS100,The prepared NPs exhibited nanoscale size distribution, narrow PDI, and a positive zeta potential, consistent with previous reports [21].WS6-loaded NPs showed a sustained release profile, which can be attributed to the release-retarding properties of the Eudragit RS100 polymer matrix. This polymer is known for its low permeability and the presence of quaternary ammonium functional groups that enable controlled diffusion of encapsulated compounds [34–36] Under the applied in vitro conditions, WS6 release is primarily governed by diffusion through the polymer network, resulting in a prolonged but partial release profile.

However, it is thought that the in vitro system does not fully recapitulate the complexity of intracellular environments. In vivo, NPs are internalized via endocytosis and trafficked into endosomal/lysosomal compartments, where acidic pH and enzymatic activity play critical roles in polymer destabilization and drug liberation. In particular, exposure to hydrolytic enzymes within the endosomal pathway can accelerate structural relaxation or partial degradation of the polymer matrix, facilitating additional drug release that is not captured under standard release conditions [37].

Furthermore, the stability study demonstrated that WS6-NPs were stable under incubation at cell culture conditions, maintaining particle sizes below 260nm with slight but not significant changes in size due to the dilution in media. In addition, PDI values remained below (0.3). While WS6-NPs initially exhibited positive charge, however after incubation in cell culture media a slight shift was observed. This shift may be explained by the adsorption of serum proteins and media components onto the WS6-NPs surface, and this phenomenon was previously reported [38]. Therefore, the surface charge alone is unlikely to direct the NPs -cell interaction under biological conditions. Moreover, small particles within the range of nanometers are internalized by endocytic pathways, including pinocytosis [39], rather than direct penetration, which may reduce membrane disruption. To evaluate their biological performance, cellular uptake was assessed by flow cytometry and confocal microscopy by preparing microfluidics cumarin-6 labeled NPs with low concentration, as tested in earlier studies [40]. The uptake exceeded 60% within the first 24hrs, demonstrating efficient cellular internalization of the WS6-NPs using Eudragit RS100 prepared by microfluidics. After the successful uptake, three main assays were used to study the viability of treated cells before differentiation: MTT assay, PI staining, and trypan blue staining, and to better observe and compare the changes over time, two time points, 24 and 72hrs were preferred to be studied among treatments. These assays confirmed the benefit of WS6-loaded NPs, As shown in Fig 2 for the MTT assay, range of concentrations (0.125–4 µM) was evaluated and the results demonstrated a clear concentration- and time-dependent effect, particularly for free WS6, which showed increased cytotoxicity at higher concentrations and longer exposure at 72hrs. In contrast, WS6-NPs exhibited enhanced cell viability among the concentration range, suggesting a protective and sustained-release effect of the NPs and based on these results, the concentration of 1 µM was selected for the subsequent experiments. Moreover, the results for PI staining conducted by flow cytometry for SCAP treated with free, unexpectedly showed a significant increase in the percentage of PI + ve cells after 24hrs compared to control (* < 0.05), indicating a rapid and early cytotoxicity of free WS6, in contrast, WS6-NPs treated group exhibited lower toxicity and this reflects the effect of treating cells with one dose at one time which aligned with previous literature showing the dose and time-dependent cytotoxic potential [41], while after 72hrs the WS6-NPs treated group showed slightly higher but not significant percentage of PI + ve cells. Importantly, this can be explained by time-dependent WS6 release. Free WS6 showed a rapid and early cytotoxic effect, as shown after 24hrs, in contrast WS6-NPs provide a sustained release profile leading to a delayed cytotoxic effect that was more obvious after 72hrs of exposure. For the trypan blue staining assay, after 24hrs a slight increase in cell count in both groups compared to the control untreated cells was observed, yet, the most significant increase was observed after 72hrs in the WS6-loaded NPs treated group, which supports the role of WS6-loaded NPs as a sustained release delivery system and a proliferation enhancer of cells. Importantly, the assays for PI staining and trypan blue staining operate through different mechanisms related to cell viability. As previously indicated, free WS6 has induced rapid and early cytotoxicity, demonstrated in the increase of PI + ve cells after 24hrs of treatment. In the trypan blue assay results, the WS6-NPs-treated group consistently showed the highest count compared to the untreated control group at all time points.

Together, these results suggest that WS6-NPs maintained better cell viability while reducing rapid and early cytotoxicity that was observed in the free WS6 group. However, WS6-NPs offer potential advantages associated with enhanced cell proliferation and survival. In addition to its proliferative effects, WS6 may exhibit immunomodulatory activity that is related to the inflammatory microenvironment observed in diabetes. In previous work, WS6-NPs significantly reduced the expression of key pro-inflammatory cytokines, including IL-6, IL-12p70, and TNF-α [42]. These cytokines are known to play central roles in insulitis and β-cell dysfunction, contributing to immune-mediated destruction and impaired insulin secretion [43]. Mechanistically, WS6 has been reported to modulate the NF-κB signaling pathway, which is a major regulator of inflammatory responses and cytokine production. Therefore, the observed downregulation of pro-inflammatory cytokines suggests that WS6-NPs may help moderate the inflammatory stress associated with the diabetic microenvironment. Furthermore, the perceptive beyond incorporating WS6 into microfluidic nanoparticles preparation was based on biocompatibility and sustained release properties of Eudragit RS100 [44]. Moreover, it was necessary to consider previous findings showing that exposure to excessive levels of mitogenic growth factors can trigger uncontrolled hyperproliferation, such as overstimulation, which has been reported to disrupt normal tissue organization and impair the regulated differentiation [45]. This assists the need for WS6-loaded NPs while differentiating SCAP cells into IPCs, which relatively reduces the need for repeated supplementation of WS6 while minimizing any toxicity. Targeted delivery strategies could substantially improve the effect of therapeutic agents designed to yield effects on beta cells or in the pancreatic environment. Delivery of such agents will result in an amplified effect for the preservation or regeneration of functional beta cells, with minimal side effects, as reported earlier [46].

For the endodermal lineage, Definitive endoderm (DE) has been successfully derived in vitro at day 7 of induction, by measuring the expression of two definitive endoderm markers, SOX17 and FOXA2, by flow cytometry and imaging system. Differentiated cells displayed an increase in the expression levels of Sox17 and Foxa2 expression consistent with definitive endoderm production. also imaging of Sox17 further validated endodermal lineage, and these results were consistent with previous literature [47].

For the maturity stage of differentiation, the expression of various markers: insulin, C-peptide, PDX-1, NKX2.2, and NKX6.1, was evaluated at day 23 of differentiation and the treatment of WS6-NPs. IPCs derived from treated groups expressed key pancreatic markers: insulin, a functional hormone secreted by β-cells, C-peptide, which confirms endogenous insulin synthesis (proinsulin) [48], PDX-1, which plays a key role in pancreas development and in β-cell function [49,50]. NKX2.2 is a homeodomain transcription factor that is critical for pancreatic endocrine cell specification and differentiation, and NKX6.1 expression is exclusive to β cells and is undetectable in other islet cells [51]. Also, recent work has shown that differentiation protocols that generate higher levels of NKX6.1 led to better outcomes for the pancreatic progenitor transplants [52]. Eventually, our data showed that all the mentioned maturation markers were expressed successfully among all treatment groups, concluding that WS6-NPs can sustain the differentiation of stem cells into IPCs by preserving the expression of their maturation markers.

After confirming the expression of β-cell maturation markers, apoptosis analysis was performed on treated groups to evaluate the effect of WS6 and WS6-loaded NPs on the differentiated cells. Apoptosis analysis revealed a significant decrease in the percentage of healthy cells (* < 0.05). On the contrary, a significant increase in the percentage of healthy cells following treatment with WS6-loaded NPs (* < 0.05) was observed, further supporting the cytoprotective role of the sustained-release formulation; however, the WS6-NPs group exhibited slightly higher but not statistically significant percentage of necrotic cells compared to the untreated control. Notably, despite the increase in the percentage of necrotic cells, WS6-NPs significantly increased the percentage of healthy cells.

After assessing apoptosis, the functionality of IPCs was further evaluated in vitro by using the glucose-stimulated insulin secretion (GSIS) assay. The GSIS cycles started with high glucose cycles to assess the instant response to glucose and the potential of the differentiated IPCs to secrete insulin. It was reported previously that differentiated IPCs exhibit immature maturation, including glucose metabolism, high basal insulin production, and limited suppression when exposed to low-glucose levels. Hence, starting with low-glucose levels may not precisely represent the actual resting state of the cells. Therefore, high glucose in the first cycle emphasizes insulin secretion despite their immature state [53]. GSIS demonstrated that differentiated IPCs among all treatment groups were responsive to alternating low (2.8mM) and high (10mM) glucose concentrations and to an SI index greater than 1, which is a fundamental criterion of the functional islet, indicating preserved glucose sensitivity [54,55]. These findings collectively demonstrate that incorporating a small-molecule stimulator of β-cell proliferation into the differentiation protocol, particularly through nanoparticle-mediated delivery, markedly improves cell viability, maintains differentiation efficiency, and functional maturation. Compared with previous reports using free small molecules or growth–factor–based differentiation alone [52]. Our approach integrates chemical modulation with nanotechnology to provide a controlled and sustained microenvironment for cell maturation, addressing a major limitation in stem-cell–derived β-cell therapy. Combining nanoparticle engineering, cellular viability, and functional validation in vitro, using a clinically relevant and readily available dental stem cell source.

Nevertheless, the long-term survival, functional stability, and glycemic control of WS6-loaded nanoparticle-treated insulin-producing cells (IPCs) still need to be confirmed in vivo because this investigation was carried out solely in vitro. Furthermore, only one WS6 concentration and exposure length were assessed, and the mechanisms underlying WS6-mediated cryoprotection and proliferation were not directly examined. Proliferative control and genetic stability are two potential long-term safety issues that were outside the purview of this study.

In order to evaluate therapeutic efficacy, durability, and safety, future research will concentrate on in vivo transplantation in diabetic models. To move this platform closer to clinical translation, additional adjustment of WS6 dose, release profile and nanoparticle design will be necessary, as will mechanistic studies of signaling pathways implicated in β-cell survival and maturation.

## 5. Conclusion

WS6 acts as a potent small-molecule stimulator of β-cell proliferation and differentiation, and its microfluidics NPs formulation represents a promising strategy to enhance the survival, functionality, and therapeutic efficacy of stem-cell–derived IPCs, advancing the goal of a cell-based alternative to insulin injection for diabetes management using NPs delivery systems for enhancing the survival rate.

## Supporting information

S1 File Graphical abstract. (TIF)
